# Effects of silymarin as adjuvant drug on serum levels of CTRP3, anti-cyclic citrullinated peptide (CCP), and high-sensitivity C-reactive protein (hs-CRP) in rheumatoid arthritis patients

**DOI:** 10.22099/mbrc.2024.48466.1876

**Published:** 2024

**Authors:** Mohammad Ehsan Elahi, Daniel Elieh-Ali-Komi, Farjam Goudarzi, Ehsan Mohammadi Noori, Shirin Assar, Mehrdad Shavandi, Amir Kiani, Homayoin Elahi

**Affiliations:** 1 Student Research Committee, Kermanshah University of Medical Sciences, Kermanshah, Iran; 2 Institute of Allergology, Charité-Universitätsmedizin Berlin, Corporate Member of Freie Universität Berlin and Humboldt-Universität zu Berlin, Berlin, Germany; 3 Fraunhofer Institute for Translational Medicine and Pharmacology ITMP, Immunology and Allergology, Berlin, Germany; 4 Regenerative Medicine Research Center (RMRC), Kermanshah University of Medical Sciences, Iran Medical Sciences; 5 Pharmaceutical Sciences Research Center, Health Institute, Kermanshah University of Kermanshah, Iran; 6 Department of Internal Medicine, Imam Reza Hospital, Kermanshah University of Medical Sciences, Kermanshah, Iran

**Keywords:** CTRP3, hs-CRP, Anti-CCP, Rheumatoid Arthritis, Silymarin

## Abstract

Silymarin is known for its anti-inflammatory and antioxidant properties. We investigated these effects on serum levels of CTRP3, Anti-CCP, and hs-CRP in individuals with Rheumatoid arthritis (RA). In this study, 42 individuals with RA were recruited and their serum specimens were collected, serum levels of hs-CRP, AntiCCP antibodies, and CTRP3 were measured using ELISA. DNA was extracted and investigated for the existence of possible new mutations in the gene encoding CTRP3 using the PCR technique; the desired fragments were then amplified and sequenced. Another blood sample was collected from the case group after taking* livergol* for three months (3 doses of 140 mg/day) and the tests were repeated. Anti-CCP Abs levels in the postintervention responding group decreased compared to preintervention (p<0.001) while in the non-responding group, the levels increased after the intervention compared to the levels before the intervention (p=0.019). Additionally, CTRP3 levels in the responding group increased postintervention (p=0.003), however, in the non-responding group the levels decreased postintervention when compared to preintervention (p=0.02). The responding group had significantly lower levels of hs-CRP when compared to that of preintervention (p=0.005) whereas the non-responding group had significantly higher levels of postintervention (p<0.001). Moreover, the results of sequencings of exon 6 on *CTRP3* gene showed the presence of mutations in exon 6 (position 215:C>T, 338:G>A, 359:A>C, and 153:T>C). Silymarin could be used as an adjuvant in the treatment of rheumatoid arthritis.

## Introduction

Rheumatoid arthritis (RA) is a chronic systemic autoimmune pathology [1]. Both innate and specific immune systems are involved in the development of the disease [2, 3]. The main symptoms include joint pain and stiffness with progressive destruction that causes disability [4]. The interplay of environmental and genetic factors that contribute to the development of RA results in a complex disease [4]. C-reactive protein (CRP) is an acute-phase protein of hepatic origin that increases in the blood following inflammation and infection [5, 6].

The test gained significance during the COVID-19 pandemic as CRP levels were notably higher in critical patients and were associated with the prognosis [7, 8]. High-sensitivity C-reactive Protein  (Hs-CRP) test measures low CRP levels up to 0.3 mg/L [9]. Anti-Citrullinated Protein (Anti-CCP) also known as anticyclic citrulline peptide is an antibody predominantly from the IgG, IgM, and IgA classes, that acts as a powerful biomarker for diagnosis of RA in the early stages [10]. In General, the presence of these Abs is associated with poorer outcomes, including boosted disease activity, radiographic progression, and disability [11]. Recently, the role of a member of the TNF-dependent protein family called C1q/TNF-related protein 3 (CTRP3), which shares a structure with adiponectin (a regulator of thermogenesis which is produced by adipocytes [12]) that is expressed and secreted in adipose tissue and monocytes, has been identified in metabolism and inflammation [13, 14]. CTRP3 is a potent anti-inflammatory adipokine that inhibits inflammatory pathways induced by fatty acids, lipopolysaccharides, and TLR ligands in adipose tissue and monocytes [12, 15]. This protein has a depressant effect on TNF and IL-6 levels [16]. Several studies have evaluated serum CTRP3 levels in patients with diabetes mellitus (DM), obesity, hypertension, and coronary artery disease. Most of these studies have reported low levels of CTRP3 in patients with heart and metabolic disease[17, 18]. In humans, the *CTRP3* gene is located on chromosome 5 (5p13.2) and has 13 exons(https://www.ncbi.nlm.nih.gov/gene/114899) [19]. Silymarin is a flavonoid compound extracted from Milk thistle (*Silybum marianum*) [20]. In various studies, the strong anti-inflammatory and antioxidant effects of silymarin have been reported [21, 22]. Since the circulating level of CTRP3 in patients with RA has yet not been evaluated, this study's main purpose was to investigate the effect of silymarin on the serum level of CTRP3 and to find possible new mutations in the *CTRP3* gene to be considered in prospective genotyping studies. 

## MATERIALS AND METHODS

**Patients recruiting:** In this study, 57 volunteer patients diagnosed with RA who had a disease history of a minimum of two years were examined by a rheumatologist after obtaining permission from the University Ethics Committee (ethics code: 23322) and were included in the study according to the criteria of the American Rheumatology Association (ACR), EULAR. 

The enrolled patients were stable RA patients under treatment of a standard drug regimen of low-dose prednisolone, hydroxychloroquine or sulfasalazine, and methotrexate. Patients with severe RA and new cases of the disease were excluded from the study, as it was possible that rheumatologists would need to change the medications or dosages during the study based on the patient's condition. Of the participants, three were excluded from the study due to dizziness, four due to stomach pain, two due to exacerbation of joint pain, and one due to bloating while discontinuing livergol. Additionally, five were excluded due to not taking the drug or irregular use of the drug from the study, and finally, 42 patients were included in the study until the end of the treatment period of which 14.3% were male, and 85.7% were female. The mean age of enrolled individuals was 47.59±12.46, and they were between the ages of 16 and 70 years old. At the beginning of the study, 10 mL of fasting blood specimen was collected of which 3 mL, was poured into an EDTA-containing tube and was used to extract DNA using the phenol-chloroform method and stored at -70°C [23, 24]. The rest of the collected blood was used to separate the serum for further analysis. The recruited patients took 140 mg livergol tablet (Goldaru-Iran) three times daily for three months, and then a blood sample was taken and retested.

**Measurement of biochemical analytes and antibody assay:** Serum hs-CRP levels were measured using a Monobind Inc ELISA kit (CA 92630, USA). Moreover, Serum CTRP3 levels were measured using a sandwich ELISA kit (DY7925-05, USAR & D System). We measured serum Anti-CCP levels using the competitive MyBioSource ELISA kit (Cat N: MBS7235871, USA) according to the manufacturer’s instructions. All ELISA tests we performed in triplicate.

**DNA extraction and genotyping:** The genomic DNA was isolated from blood leukocytes using the phenol-chloroform method (TaiwanVIOGENE) [24, 25]. The majority of the detected mRNA (approximately 76%) was linked to exon 6 of the *C1QTNF3 (CTRP3*) gene, as shown in the NCBI Nucleotide report (*https://www.ncbi.nlm.nih.gov/nuccore/NM_181435.6?report= graph*).  Additionally, crucial regulatory regions, including the PolyA, are located within exon 6. Therefore, exon 6 was selected as the primary target for sequencing. In contrast, the other exons of the C1QTNF3 gene were relatively short in length, which made it challenging to effectively amplify and sequence using PCR. As a result, the focus was placed on thoroughly analyzing exon 6, which contained the most abundant mRNA transcripts and important regulatory elements.

PCR technique was used to amplify exon 6 of the gene encoding *CTRP3*. Two pairs of primers were designed based on the sequence in GeneBank and using the primer3 online Website (*https://primer3.ut.ee/*) to amplify 715bp and 855bp components (Table 1). To serve as controls, we utilized DNA samples from local healthy individuals without any history or symptoms of rheumatologic conditions. The PCR reaction was performed using a 25 uL volume of PCR reaction mixture. The PCR process was performed in 35 cycles using the temperature cycle. The product of PCR was electrophoresed on 2% agarose gel[23]. After the amplification of the desired fragments, the PCR product was sequenced using the Sanger method to identify possible new mutations in exon 6 of the *CTRP3* gene. Sequencing was performed using ABI 3730 capillary sequencer (Life Technologies, Carlsbad, CA) and the results were analyzed using SnapGene 5.0.8 software.

**Table 1:** Primer sequence, length of the piece, and PCR condition considered for amplification of *CTRP3 *gene 715 and 855 bp components.

**Gene**	**Primer sequence**	**Product size**	**Temperature program**
*CTRP3*	Forward: 5`GGTCAGGAGGTTGAGGTTCA3’ Reverse: 5'TTTTAGCATCTCAGTTTTGGGACAC3'	715 bp	95°C, 5 min; 95°C, 30 sec; 59°C, 30 sec; 72°C, 40 sec; 72°C, 10 min; 35 cycles
*CTRP3*	Forward:5`GTGTCCCAAAACTGAGATGCTAAAA3' Reverse: 5'CTTAGCCCTTTGCCTTCTATG3'	855 bp	95°C, 5 min; 95°C, 30 sec; 59°C, 30 sec; 72°C, 40 sec; 72°C, 10 min; 35 cycles

**Note:** Primer Forward (10pmol): 1 μL, Primer Reverse(10pmol): 1 μL, MgCl_2_(50Mm): 1 μL, PCR Buffer10X:2.5, dNTPs (200 μM): 0.5μL, ddH2O: 19.05 μL, Taq DNA Polymerase (5 U/μL):0.2 μL, DNA: 1 μLdNTPs (SinaClon, Cat No: MM2082)- Taq DNA Polymerase (SinaClon, Cat No: DP1601)

**Statistical Analysis: **The frequency distribution of qualitative data in the study group was performed using the Chi-square test (χ2). The normal distribution of quantitative variables was investigated using the One-Sample Kolmogorov-Smirnov test. Mean serum levels of CTRP3, hsCRP, and anti-CCP were compared before and after receiving livergol in patients with RA using a Paired Sample T-Test. SPSS16 (Ver 16, SPSS Inc., Chicago, USA)) was used for data analysis, and P<0.05 was considered statistically significant.

## Results

In this study, according to the intervention and the effectiveness of silymarin on a specific subgroup of patients, we divided the individuals into two responding and non-responding groups. In the responding group, CTRP3 levels were increased, but anti-CCP and hs-CRP levels were decreased. Patients in the non-responding group were found to have decreased CTRP3 levels, but increased levels of anti-CCP and hs-CRP levels. The mean serum level of anti-CCP Abs in the postintervention responding group decreased significantly compared to preintervention (96.70±90.16 vs 134.40±118.06 respectively; p<0.001) while in the non-responding group, the levels increased after the intervention compared to the levels before the intervention (129.83±55.02 vs 116.65±51.22 respectively; p=0.019). 

**Figure 1:**
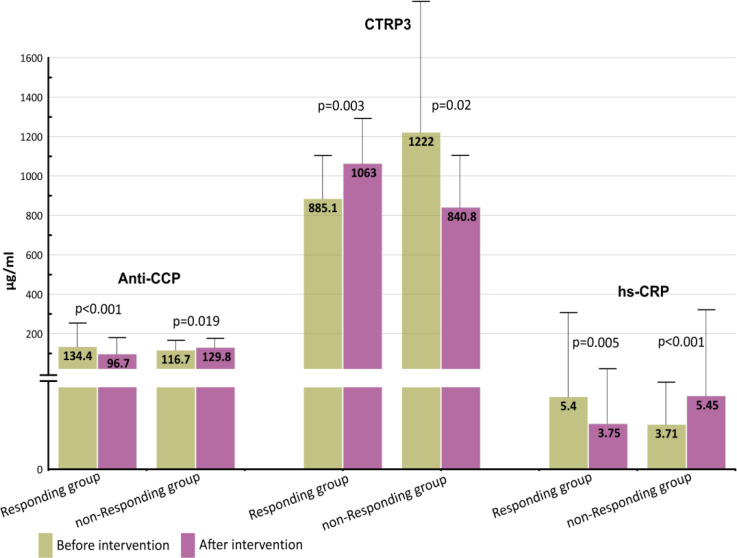
Comparison of mean serum levels of anti-CCP Abs, CTRP3, and hs-CRP levels in responding and non-responding subgroups.

The mean serum CTRP3 level in the response group increased after treatment compared to levels measured before treatment (1062.7±248.60 vs 885.15±227.6; p=0.003), however, in the non-responding group the mean serum level of CTRP3 decreased after intervention in comparison to the levels reported before intervention (840.79±282.70 vs 1222±669.38; p=0.02). Assessment of hs-CRP revealed that the responder group had significantly lower levels of hs-CRP when compared to the levels measured before livergol treatment (3.7 ±3.61 vs 5.40±5.35; p=0.005) whereas the non-responding group was found with significantly higher levels of hs-CRP after treatment 5.45±5.39 vs 3.71±2.61; p< 0.001). (Fig. 1, Table 2).

**Figure 2:**
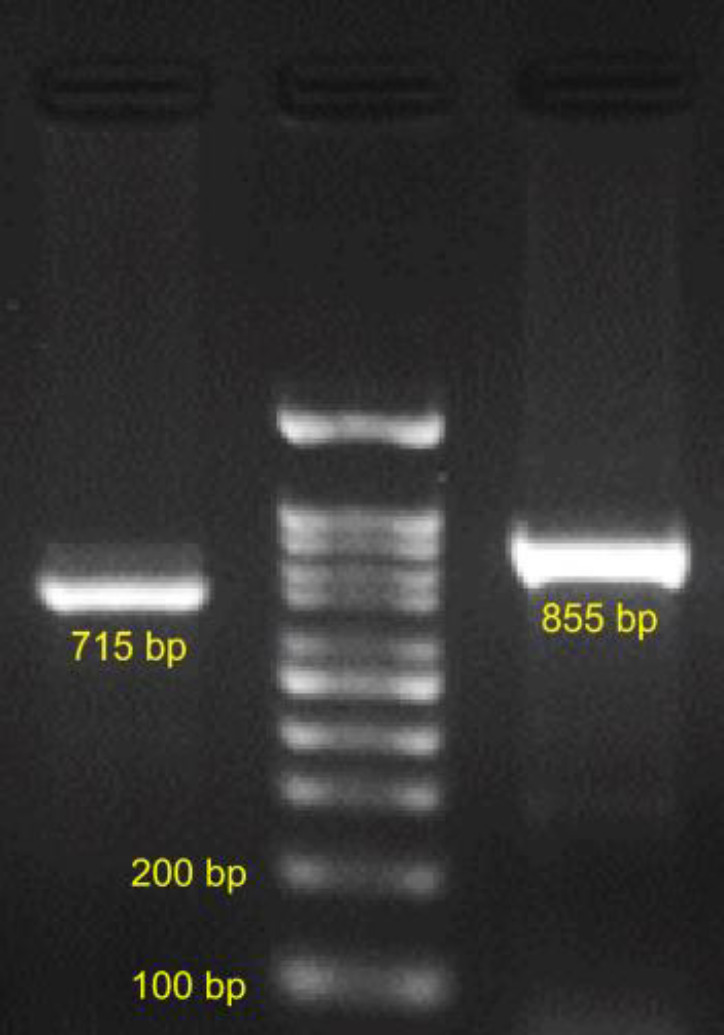
The electrophoresis pattern of PCR product resulting from exon 6 amplification of *CTRP3* gene.

**Figure 3:**
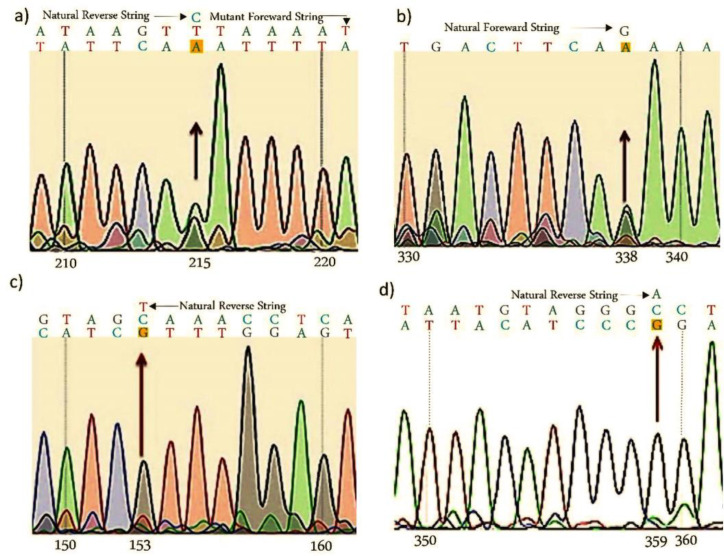
Exon 6 sequencing results of genes encoding CTRP3) a: position 215:C>T, b) 338:G>A, c) 153:T>C, and d) 359:A>C.

The electrophoresis pattern of exon 6 on *CTRP3* is represented in Figure 2. According to the figure, two bonds are formed, 715 bp and 855 bp. Figure 3 represents the data obtained from sequencing of exon 6 on *CTRP3* to determine mutations. According to Figure 3a, in the presented section, the nucleotide on the reverse strand in position 215 (green peak) is A therefore, the complementary nucleotide on the forward strand would be T. Since the wild type of nucleotide in this point is C, therefore, the mutation is C to T (C>T). In Figure 3b the nucleotide in position 338 on the forward strand (green peak) of the patient is A, therefore, the wild-type allele is G and the mutation is G>A. Similarly, Figure 3c is a section of the reverse strand, the nucleotide on the reverse strand in position 153 (gray peak) is G therefore, the complementary nucleotide on the forward strand is C and the wild-type allele is T, therefore the mutation is T>C. Figure 3d is a section of the reverse strand, the nucleotide on the reverse strand in position 359 is G therefore, the complementary nucleotide on the forward strand is C and the wild-type allele is A, therefore the mutation is A>C. Table 3, represents the position of newly identified mutations.

**Table 2:** Comparison of mean ± SD serum levels of anti-CCP ab, CTRP3, and hs-CRP levels in the patient group before and after treatment (response and no response to treatment).

	**Before intervention**	**After intervention**	**P-value**
**Anti-CCP**			
Responding group (n=29) ± SD	134.40 ± 118.06	96.70 ± 90.16	<0.001
Non-responding group (n=8)	116.65 ± 51.22	129.83 ± 55.02	0.019
**CTRP3**			
Responding group (n=14)	885.15 ± 227.6	1062.7 ± 248.60	0.003
Non-responding group (n=24)	1222± 669.38	840.79 ± 282.70	0.02
**hs-CRP**			
Responding group (n=15)	5.40 ± 5.35	3.75 ± 3.61	0.005
Non-responding group (n=22)	3.71 ± 2.61	5.45 ± 5.39	<0.001

**Table 3:** Mutations detected in CTRP3 coding gene (exon 6)

**Position**		**Type of mutation**	**Exon **
Codon 215		C>T	Exon 6
Codon 338		T>C	Exon 6
Codon 259		G>C	Exon 6
Codon 153		A>C	Exon 6

## Discussion

Rheumatoid arthritis is a chronic systemic autoimmune disease that first affects the joints and may have devastating effects on the joints and other organs [26, 27]. Genetic and environmental factors can cause disease progression [28]. 

CRP is a 23 kD pentamer protein that is secreted by liver cells in response to a variety of inflammatory cytokines such as IL-1, IL-6, and TNF [29, 30]. Serum CRP levels are commonly used in rheumatoid arthritis (RA) as an alternative marker of systemic inflammation [5]. The high-sensitivity assays for CRP enable the detection of extremely low concentrations of the biomarker [31]. CTRP3 in adult mice is known as adiponectin produced by adipose tissue and is involved in energy storage and metabolism. It is also produced in other tissues of organs such as the liver, lung, testicle, heart, etc., which indicates a wide range of producing sources and its possible roles in these sites. Its roles include maintaining the function of the cardiovascular system, regulation of inflammation, glucose/lipid metabolism, etc [32]. In this study, we investigated its role in inflammation. CTRP3 is an anti-inflammatory agent that is inversely related to inflammatory cytokines such as IL-1, IL-6, etc. It has also been observed to have great potential for treating some inflammatory diseases such as IgA nephropathy (IgAN) [13, 33]. Based on these data, we expect CTRP3 to be effective in the treatment of rheumatoid arthritis.

This study aimed to use the anti-inflammatory properties of silymarin to reduce inflammation caused by rheumatoid arthritis determined by reducing anti-CCP and hs-CRP inflammatory factors and possibly increase the anti-inflammatory factor CTRP3, which is thought to have a protective role in rheumatoid arthritis. Weight et al. 2005 and Kopp et al. 2010 showed that CTRP3 has anti-inflammatory properties by inhibiting cytokines produced by human monocytes [14, 34]. When CTRP3 levels were increased, the incidence of disease in these individuals decreased significantly [19, 32]. The results of this study are consistent with the present study. 

Anti-CCP antibodies are autoimmune antibodies directed against peptides and citrulline proteins. They are the second serological markers in the ACR / EULAR criteria for early diagnosis and treatment of rheumatoid arthritis[9, 35]. Citrulled anti-protein antibodies are antibodies that are made against the individual's citrullinated peptides and proteins. These antibodies are found in most people with rheumatoid arthritis. Clinically, CCPs are used to detect these antibodies in the blood or serum [9]. Since anti-CCP is one of the specific factors in rheumatoid arthritis, it is used to diagnose the causes of the disease [34]. The results of Papadopoulosthe et al showed that the presence of aCCP auto-Abs is linked to more active and severe manifestation in terms of the time of diagnosis and during the disease course, however, serum levels of anti-CCP do not necessarily correlate with disease manifestation and activity [33]. In a 2015 study by Ebrahimpour Kojan et al., on 40 patients with type 2 diabetes mellitus, it was reported that silymarin can reduce hs-CRP levels by 26.83% compared with the placebo group. The result of the present study is consistent with the above study [36]. In another study performed by Abdul Rahman Hussein et al., on 60 patients (30 men and 30 women) with rheumatoid arthritis in Iraq, it was shown that Silybinin could lower anti-CCP and hs-CRP levels in patients with rheumatoid arthritis [37]. The results of our study are consistent with this study. Additionally, in a study by Elgarf et al., on 40 patients with type 2 diabetes mellitus in Egypt, the results of the study indicated a significant decrease in hs-CRP levels [38]. The results of our study are consistent with this study. In another research by Derosa et al., on 143 patients with dyslipidemia, silymarin was shown to be effective in reducing the inflammatory factor hs-CRP[39]. According to the results presented in Table 2 , the levels of inflammatory factors hs-CRP and anti-CCP, which had increased in the patient group before the intervention, decreased after the intervention with the anti-inflammatory effect of silymarin; Also, the level of anti-inflammatory factor CTRP3 increased after the intervention. 

The main limitation of the present study is the low number of the recruited individuals. We recommend including more individuals to have a better statistical analysis. Moreover, we also recommend adding RF and antinuclear antibody (ANA) tests to the testing parameters. Another limitation of this study was the lack of data on a correlation between each allele (protective and risk) for each described mutation with the serum levels of hs-CRP, anti-CCP antibodies, and CTRP3.

## Conflict of Interest:

The authors declare that they have no conflict of interest.

## Authors’ Contribution:

Conceptualization: AK, DEAK; Methodology: MEE, DEAK, FG, EMN, SA, MSH; Formal analysis and investigation: AK, HE; Writing - original draft preparation; Writing - review and editing: DEAK, AK; Supervision: AK; Graphic work: DEAK.
